# Of Cephalic Version in Shoulder Presentation with the Arm in the Vagina

**Published:** 1855-07

**Authors:** R. A. F. Penrose


### *Of Cephalic Version in Shoulder Presentation with the Arm in the Vagina.* By R. A. F. Penrose, M.D.

(As read before the College of Physicians.)

The case to which I wish to call the attention of the College, is interesting, as having been one of difficult labor, in which an unusual method of delivery was attempted and entirely succeeded. The case to which I refer was one of shoulder presentation, and the treatment consisted in bringing the *head* of the child to the superior strait, instead, as is almost universally recommended, making version by the feet.

My attention had been called to this plan of treatment for similar cases by a pamphlet of Dr. Wright, of Cincinnati, in which he advocates the resort to version by the head in all cases where the shoulder presents, even though the arm of the child be in the vagina.

It is not necessary that I should ocupy the time of the college in referring to the opinions of authors on this subject, as they are doubtless well known to all present; the almost universal direction in these cases being, particularly when the arm is in the vagina, to resort to version by the *feet.*

The following is a history of the case which I take from my notes:—

On the evening of the 3d of April I was called, by a midwife, to see Mrs. M——, as a case of difficult labor which she could not manage. On reaching the patient I learned that she had been taken in labor some time in the morning; that at two o'clock she had given birth to a child, and that the midwife, on making an examination, discovered a second child, presenting by the shoulder, its arm being in the vagina. This was at two o'clock in the afternoon. I did not see the patient until about seven o'clock, five hours after the rupture of the membranes. I then found her with the uterus contracted spasmodically upon the child, suffering a great deal of pain in consequence, whilst her condition had not been at all improved by brandy freely given by the midwife.

On making an examination, I found the arm in the vagina, considerably swollen, the shoulder at the superior strait, the head high up in the right iliac fossa, being readily felt through the abdominal walls, the back of the child towards the symphysis pubis. Having carefully diagnosed the position of the child, I determined to resort to version by the *feet*, but met with so much difficulty from the firm contraction of the uterus, together with the great irritability and restlessness of the patient, who rolled and struggled so violently when I attempted the operation, partly no doubt from the effects of the brandy she had taken, that I was forced to desist. I then determined to try the plan of treatment as suggested by Dr. Wright, which consisted, as the first step, in carefully returning the arm into the cavity of the uterus, which was not very difficult, and then, with two fingers on the shoulder I endeavored, not so much to push it up, as to slide it away from the superior strait, attempting, in fact, to imitate spontaneous version, by causing the whole body of the child to revolve in the cavity of the uterus; this action upon the shoulder was very much facilitated by an external manipulation, through the abdominal walls, upon the head in the iliac fossa, which consisted in attempting to force it toward the superior strait. To my surprise the shoulder readily yielded and ascended; the next minute, still continuing the process, the neck, and shortly after the side of the head, were felt at the superior strait, and then, by a little more manipulation, the vertex was brought down in the left occipito-anterior position. The moment the head was placed in the proper position, the patient expressed herself completely relieved from the severe pain she had been suffering for hours.

For a time, the uterus ceased entirely to act, but pains coming on they were stimulated by ergot, the parts having been dilated by the passage of the first child, and, in about an hour after, the child was born ALIVE.

It is proper, however, to state, as explaining, to a certain extent, the facility with which the operation was performed, that the child weighed only five pounds, yet on the other hand it should be recollected that the smallness and mobility of the child is perhaps the most common cause of shoulder presentations, and hence, in such presentations, we may expect, very generally, to have small children to deal with, and moreover that version by the *feet* had been attempted, and had not been accomplished.

It might be urged that, as the child was not large, the case might have been left to nature, and labor terminated by spontaneous evolution. But what are the facts of the case? Five hours had elapsed since the rupture of the membranes, and one of the earliest changes noticed when spontaneous evolution takes place had not yet occurred; I refer to the rotation of the presenting shoulder to the symphysis pubis, as preparatory to its engagement beneath the arch; indeed, the spontaneous expulsion of the child in these cases is the exception, not the rule; and when it does happen, if we think for a moment on the mechanism of labor by which delivery is accomplished, we cannot fail to perceive that the mother must be exposed to all the dangers attending a long and painful labor, while the child is so powerfully compressed that death is the general result. Velpeau gives statistics of one hundred and thirty seven cases of spontaneous evolution, in which but twelve children were born alive.

It is asserted by some that an error of diagnosis has existed in those cases where it is stated that version by the vertex has been successfully performed in shoulder presentations; that they have been really cases of head presentation, complicated by a descent of the arm. But this certainly was not so in the case to which I refer, the head being distinctly felt in the right iliac fossa, through the abdominal walls, whilst the partial introduction of the hand into the cavity of the uterus, in the attempt to make version by the feet, enabled me perfectly to recognize the position of the child.

Again, it is said that the operation of version by the vertex is a very difficult one, that we cannot, without subjecting the mother to very great danger, push up the presenting part of the child, and search for the head; and then, having found it, that we cannot grasp the round and slippery occiput and bring it to the superior strait. This objection in many cases may be well founded, but the operation which I have related consisted, rather, in pushing the shoulder away from the superior strait, causing, thereby, the child to revolve in the cavity of the uterus; of course as soon as such a motion of rotation is communicated to it, by pushing away the shoulder, we must, of necessity, have some other part brought to the superior strait, which part will be first the neck, then the head, and by a little manipulation the occiput can be brought down, and an *impracticable* case of labor can be thus changed, without, or with comparatively little pain to the patient, and in a few minutes, to one of the most simple character; and not only thus changed, but effected without exposing the child to the great risks to which it is always liable when version by the feet is resorted to; and, if an attempt to make version by the vertex should fail, without giving our patient any more pain, our hand being in the vagina, our fingers in the mouth of the uterus, we can most easily, if the case admit of it, pass our hand upwards, grasp the feet, and bring them down.

